# Decision-Making Underlying Support-Searching in Pea Plants

**DOI:** 10.3390/plants12081597

**Published:** 2023-04-10

**Authors:** Qiuran Wang, Silvia Guerra, Bianca Bonato, Valentina Simonetti, Maria Bulgheroni, Umberto Castiello

**Affiliations:** 1 Department of General Psychology, University of Padova, 35131 Padova, Italy; silvia.guerra@unipd.it (S.G.); bianca.bonato.1@phd.unipd.it (B.B.); valentinasimonetti@ab-acus.eu (V.S.); umberto.castiello@unipd.it (U.C.); 2 Ab.Acus srl, 20155 Milan, Italy; mariabulgheroni@ab-acus.com

**Keywords:** decision-making, plant movement, kinematics, plant behavior

## Abstract

Finding a suitable support is a key process in the life history of climbing plants. Those that find a suitable support have greater performance and fitness than those that remain prostrate. Numerous studies on climbing plant behavior have elucidated the mechanistic details of support-searching and attachment. Far fewer studies have addressed the ecological significance of support-searching behavior and the factors that affect it. Among these, the diameter of supports influences their suitability. When the support diameter increases beyond some point, climbing plants are unable to maintain tensional forces and therefore lose attachment to the trellis. Here, we further investigate this issue by placing pea plants (*Pisum sativum* L.) in the situation of choosing between supports of different diameters while their movement was recorded by means of a three-dimensional motion analysis system. The results indicate that the way pea plants move can vary depending on whether they are presented with one or two potential supports. Furthermore, when presented with a choice between thin and thick supports, the plants showed a distinct preference for the former than the latter. The present findings shed further light on how climbing plants make decisions regarding support-searching and provide evidence that plants adopt one of several alternative plastic responses in a way that optimally corresponds to environmental scenarios.

## 1. Introduction

Scientists have long been intrigued by the specialized adaptations of climbing plants that enable them to compete for necessary resources, such as sunlight [1]. However, despite this prolonged fascination, we know surprisingly little about how climbing plants make ‘decisions’ with regard to stimulus searching and attachment behaviors. Indeed, climbing plants can be an ideal model system for studying the decision-making in plants because they show rapid changes in response to environmental cues [2]. For them, finding a suitable support upon which they can climb is among the most important factors affecting their growth and development [3].

The study of climbing plant behavior is chiefly based on Darwin’s observations on the oscillatory movements of exploring stems and tendrils (i.e., *circumnutation*) [4]. He noted that vines are not only able to locate potential supports and grow towards them, but they can even show an aversive response [4]. He first described this effect with regard to the *Bignonia capreolata* L. tendrils that initially seized and then let go of sticks that were inappropriate in terms of size. If, because of its thickness, a stimulus was perceived as ‘inadequate’, after initially seizing it, the tendrils let go of it [4]. This case provides a degree of support to speculative claims that some climbing plants can judge the thickness of potential supports and modify their circumnutation patterns to a greater or lesser extent, depending on the features of potential supports with respect to what would be expected by chance movement. To date, most studies on climbing plants focus on the attachment stage, which is the final coiling step [5], whereas the approaching stage, which occurs before any physical contact with the support, is rarely examined. Experimental evidence demonstrates that this stage can be anticipatory and adapted on the basis of the physical properties of the support, and hence it has the potential to be highly informative [6,7,8,9,10]. For instance, Guerra and colleagues demonstrated that pea plants (*Pisum sativum* L.) are able to perceive a support and modulate the kinematics of the tendrils’ aperture depending on its thickness [8]. The aperture of the tendrils refers to the maximum distance between the tips of the tendrils reached during movements leaning towards a support. The average and maximum velocities of the tendrils were found to be higher for thinner supports compared to thicker ones. In temporal terms, it took more time for the tendrils to reach peak velocity and maximum aperture when the supports were thinner [6,8]. Further, they modulate the production of a number of secondary velocity peaks (i.e., submovements) as a function of the support’s thickness, suggestive of “on-line adjustments” [7]. The frequency of submovements tends to increase when the support is thick. This signifies that they need to make more adjustments in order to establish contact points along the support [7].

The above results are in line with Darwin’s previous observations highlighting that thinner and thicker supports are different for climbing plants [4,5,11,12,13], with the touching and grasping of thick supports being more ‘difficult’ since it is more energy-demanding with respect to the thinner ones. In fact, it implies that the plant not only needs to increase the length of its tendrils in order to wrap itself around the stimulus efficiently [14], but it also has to strengthen its tensional forces to counteract gravity [2,15] and modulate kinematics [8]. Still, could climbing plants “choose” between thinner and thicker supports? Should they manifest a preference for the thinner? Would they perform according to their choice?

In light of these considerations, the aim of the current study is twofold. First, to ascertain what pea plants do when confronted with differently sized supports. To test this, after germination, pea plants were exposed to both a thin and thick support. We hypothesized that if pea plants inevitably prefer thinner supports, then we should observe a significantly higher frequency of movements directed toward them. Second, to ascertain whether such a decisional process impacts the kinematics of the tendrils’ circumnutations, we compared a ‘choice’ condition termed the “double-support” (DS) condition, in which a thin and thick support were present in the environment with a “single-support” (SS) condition, where only a thin support was present in the environment. We foresee differences across the conditions evident at the level of movement kinematics. Although the plants would prefer the thinner support, they might still keep into account the thicker one as a potential option for an ever-changing environment. If so, a hybrid kinematical patterning accounting for differently sized supports would be evident.

## 2. Results

### 2.1. Qualitative Results

**Figure 1:**
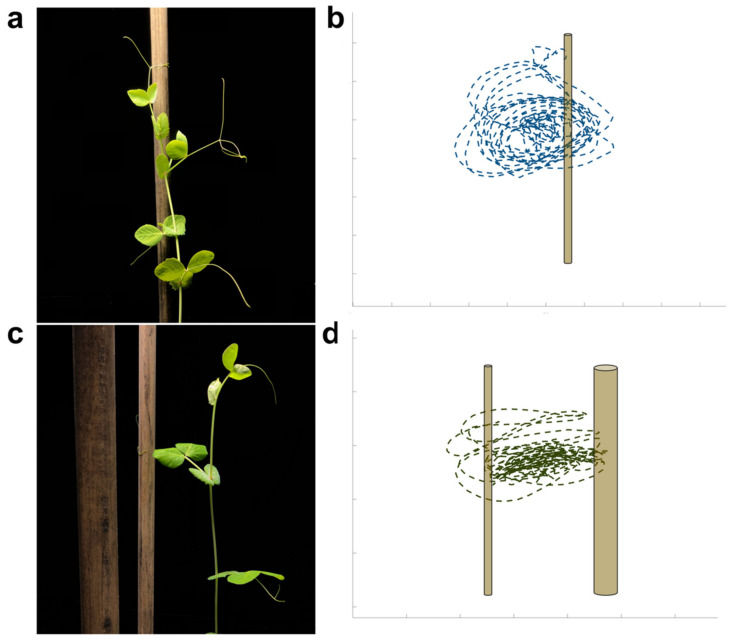
A frame representing an exemplar plant approaching the support for (**a**) the single-support (SS) condition with (**b**) a graphical representation of its trajectory. A plant approaching the thinner support for (**c**) the double-support (DS) condition with (**d**) a graphical representation of its trajectory.

For all plants and in both experimental conditions (i.e., DS and SS), the tendrils displayed a circumnutating growing pattern. As soon as a plant sensed the support, it strategically altered the tendril’s movement trajectory so as to bend towards the support (Figure 1a,b). For the DS condition, plants exhibited a very strong preference for the thin support and grew less than the plants for the SS condition by the time they touched the support (Figure 1c,d). Eight of the nine plants for the DS condition began to grow and move toward the thin support relatively early, even though they were too tiny to reach out for any support. These plants were able to aim fairly precisely toward the thin support and touch it by modulating/twisting the angles of the new petiole, and this is visible to the naked eye (see Video S1). Only one plant made an attempt to cling onto the thick support but ultimately failed and fell. The data for this plant have not been analyzed further. In this connection, given that we could not observe any full movement towards the thicker support for the DS condition, here, we consider only the thin SS condition as a comparison with the DS condition.

Among the eight plants in the SS condition: two circumnutated clockwise and two circumnutated counterclockwise. The remaining four exhibited both a clockwise and counterclockwise circumnutation pattern during the entire movement. As for the DS condition, four plants circumnutated clockwise, one plant circumnutated counterclockwise and three circumnutated in a mixed manner.

### 2.2. Kinematic Results

The descriptive statistics and the kinematic results, when comparing the DS with the SS conditions, are provided below (Table 1 and Table 2). The comparison is between the thin support for the SS condition and the thin support for the DS condition. This is because, for the DS condition, plants always choose the thinner support.

#### 2.2.1. Number of Circumnutations

For the DS condition, subjects performed, on average, 24.924 (SD = 4.247, SE = 0.303, 95% CI: [24.327, 25.521]) circumnutations, whereas for the SS condition, they performed, on average, 26.553 (SD = 6.156, SE = 0.439, 95% CI: [25.688, 27.418]) circumnutations. The Bayesian Mann–Whitney U analysis revealed a Bayes factor (BF_10_) of 314.656, suggesting that there is a decisive difference between the SS and the DS conditions with respect to the number of circumnutations (BF_10_ = 314.656, BF_01_ = 0.003, W = 14220, R-hat = 1.008, 95% CI: [−0.657, −0.229]).

#### 2.2.2. Circumnutation Duration

The duration of the circumnutation was, on average, 66.746 min for a single circumnutation (SD = 13.190, SE = 0.940, 95% CI: [64.893, 68.600]) for the DS condition, whereas for the SS condition, it was, on average, 69 min (SD = 14.451, SE = 1.030, 95% CI: [66.969, 71.031]). The Bayesian Mann–Whitney U analysis revealed a Bayes factor (BF_10_) of 0.387, suggesting that there is no difference between the SS and the DS conditions with respect to the circumnutation duration (BF_10_ = 0.387, BF_01_ = 2.584, W = 17083, R-hat = 1.000, 95% CI: [−0.354, 0.029]).

#### 2.2.3. Distance from the Circumnutation Gravity Center to the Origin

The distance from the circumnutation gravity center to the origin was 15.899 cm (SD = 10.429, SE = 0.743, 95% CI: [14.434, 17.364]) for the DS condition, whereas it was 27.895 cm (SD = 24.340, SE = 1.734, 95% CI: [24.475, 31.315]) for the SS condition. The Bayesian Mann–Whitney U analysis revealed a Bayes factor (BF_10_) of 136.096, suggesting that there is a decisive difference between the SS and the DS conditions with respect to the distance from the circumnutation gravity center to the origin (BF_10_ = 136.096, BF_01_ = 0.007, W = 15132, R-hat = 1.031, 95% CI: [−0.575, −0.169].

#### 2.2.4. Length of the Circumnutation Major Axis

The length of the circumnutation major axis was 91.214 mm (SD = 38.929, SE = 2.774, 95% CI: [85.744, 96.684]) for the DS condition, whereas it was 72.908 mm (SD = 43.538, SE = 3.102, 95% CI: [66.791, 79.026]) for the SS condition. The Bayesian Mann–Whitney U analysis revealed a Bayes factor (BF_10_) of 734.705, suggesting that there is a decisive difference between the SS and the DS conditions with respect to the length of the circumnutation major axis (BF_10_ = 734.705, BF_01_ = 0.001, W = 24455, R-hat = 1.016, 95% CI: [0.275, 0.676]).

#### 2.2.5. Circumnutation Length

The circumnutation length for the DS condition was 243.403 mm (SD = 124.957, SE = 8.903, 95% CI: [225.846, 260.961]), whereas, for the SS condition, it was 188.148 mm (SD = 115.972, SE = 8.263, 95% CI: [171.853, 204.443]). The Bayesian Mann–Whitney U analysis revealed a Bayes factor (BF_10_) of 980.421, suggesting that there is a decisive difference between the SS and DS conditions with respect to the circumnutation length (BF_10_ = 980.421, BF_01_ = 0.001, W = 24433, R-hat = 1.015, 95% CI: [0.290, 0.693]).

#### 2.2.6. Circumnutation Area

The area of circumnutation for the DS condition is, on average, 4992.504 mm^2^ (SD = 4634.422, SE = 330.189, 95% CI: [4341.325, 5643.684]), whereas for the SS condition is 3217.099 mm^2^ (SD = 3505.097, SE = 249.728, 95% CI: [2724.601, 3709.598]). The Bayesian Mann–Whitney U analysis revealed a Bayes factor (BF10) of 1267.886, suggesting that there is a decisive difference between the SS and DS conditions with respect to the area of circumnutation (BF_10_ = 1267.886, BF_01_ = 0.0008, W = 24611.5, R-hat = 1.008, 95% CI: [0.299, 0.697]).

#### 2.2.7. Amplitude of Maximum Peak Velocity

The amplitude of maximum peak velocity was, on average, 6.541 mm/min (SD = 5.650, SE = 0.403, 95% CI: [5.748, 7.335]) for the DS condition, whereas it was 4.660 mm/min (SD = 2.840, SE = 0.202, 95% CI: [4.260, 5.059]) for the SS condition. The Bayesian Mann–Whitney U analysis revealed a Bayes factor (BF_10_) of 4137.588, suggesting that there is a decisive difference between the SS and DS conditions with respect to the amplitude of maximum peak velocity (BF_10_ = 4137.588, BF_01_ = 0.0002, W = 25438, R-hat = 1.014, 95% CI: [0.380, 0.780]).

#### 2.2.8. Correlational Analyses

**Figure 2:**
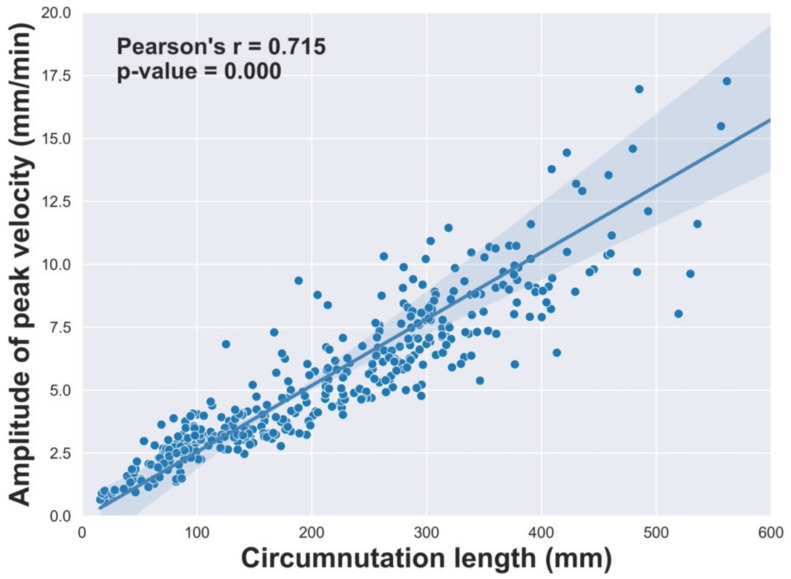
Pearson’s correlation coefficient between the “circumnutation length” and the “amplitude of peak velocity”.

We noticed a non-significant difference for the circumnutation duration across conditions, while the amplitude of peak velocity increased for the DS with respect to the SS condition. We felt that this might indicate the plants putting in place a sort of isochrony principle [16] (see the Discussion section). To test this, we performed Pearson’s correlation analysis [17] between the circumnutation length and the amplitude of peak velocity [18]. The results indicate a significant correlation between these measures (Pearson’s *r* = 0.715, *p*-value = 0.000, 95% CI: [0.663, 0.760]; Figure 2).

## 3. Discussion

In this study, we have examined the kinematics of pea plants’ tendrils’ circumnutation from the beginning of circumnutation till they touched and grasped the support. Our findings show that most of the considered dependent measures differed markedly between the DS and SS conditions, indicating that pea plants exhibit distinct movement patterns depending on the conditions. For instance, plants perform fewer and larger circumnutations, as evidenced by a lower “number of circumnutations”, a longer “length of circumnutation major axis”, and a longer “circumnutation length” for the DS than the SS condition. Further, the “circumnutation area” is greater for the DS than the SS condition. To achieve all this, plants increased the “amplitude of maximum peak velocity” for the DS condition. Altogether, this pattern of results might imply a more active and exploratory patterning for the plants facing a “choice” scenario. The “circumnutation duration”, on the other hand, remains the same for both conditions. In this respect, the correlational analysis indicates that the “circumnutation length” and the “amplitude of the peak velocity” are strongly correlated. This suggests that the pea plants’ movement is based on the isochrony principle [16]. The isochrony principle refers to a spontaneous tendency to increase the velocity of a movement depending on the linear extent of its trajectory to maintain the execution time as approximately constant [19]. In our circumstances, plants maintain constant movement duration and scale velocity in order to cover longer distances, as witnessed by the longer circumnutation lengths. This appears to be an easy and appropriate organizational option adopted by the plant to program the patterning of circumnutation when a decision based on alternatives has to be taken.

At this stage, the question is more about how climbing plants avoid an unsuitable host and choose a suitable one. A common belief is that the physiological mechanisms underlying behavioral responses in plants tend to be caused by simple, local reactions [20]. As proposed by Saito, these ‘reactions’ might also be the basis of the decision-making processes related to the support diameter characterizing tendrils’ coiling [5]. In this view, changes in the coiling responses may be caused by local reactions in the tendrils. For instance, in many climbing plants, the coiling of tendrils is thought to be caused by the contraction of the gelatinous fibers (G fibers) after stimuli have been contacted [5,21]. That is to say, when a suitable support is detected and recognized, the tendril shows a reflex behavior and rapidly bends in the stimulated direction [22]. Put simply, at the basis of plants’ support selection, there might be a mechanism that makes it possible to select a support with an appropriate diameter.

The emerging picture from the “choice” that the plants made might suggest a trade-off in terms of metabolic use. Touching and grasping a thicker support would imply the growth of longer tendrils, which, in turn, would be more demanding in terms of energy exploitation. This metabolically based decision would also reflect on movement kinematics. The movement towards thicker supports is much slower than for thinner supports [8] and shows a great deal of online adjustments, visible as submovements along the velocity profiles [7]. Therefore, plants might have the ability to monitor, detect, and process information that determines the preference for a thin support. These aspects are particularly evident when comparing circumnutation between the thin support for the SS and the DS conditions. Plants move faster and execute less but larger circumnutations for the latter than for the former. This signifies that, despite that the plants are aiming at supports of the same size, being exposed to an alternative (the thicker support for the DS condition) determines a decisional complexity that is played out in the kinematics of circumnutation. Therefore, it appears that circumnutation is not only affected by a complex occurrence of factors, such as light, gravity, touch, and hormonal signals [23], but also by the presence of alternative supports in the environment.

Decision-making implies making choices from several alternatives to achieve a desired result [24]. In recent years, decision-making has been studied on a variety of organisms [25], including plants [26,27]. Dener and colleagues investigated decision-making in the root development of the pea plant (*Pisum sativum*) using the risk sensitivity theory (RST) [26]. According to RST, the rational decision is the one that maximizes fitness [28]. In the study, root growth displayed both risk-prone and risk-averse behaviors, which better support the RST hypothesis than previous animal testing. It appears that pea plants make “rational” economic decisions in terms of risk sensitivity [26,29]. Plant decision-making is also explored in the context of the social environment. Gruntman and colleagues compared the responses of *Potentilla reptans*, centered on their ability to out-compete their neighbors for accessing light [27]. Observed shifts in the responses between vertical growth, shade tolerance, and lateral growth suggest that plants can choose adaptively from several alternatives under light-competition scenarios [27].

Altogether, these findings suggest that plants possess the ability to make decisions and adjust their behavior in response to their surroundings. Our findings contribute to the literature, demonstrating that a plant’s behavior is flexible, as opposed to rigid and mechanical [30], reinforcing the idea that plants are systems with a remarkable ability to deal with the complexities of an ever-changing environment [31].

At this stage, the question is how and at which level pea plants implement such decisions that then translate into specific behavioral patterns. One possible mechanism could be light acquisition at the level of the stomata [32,33], which might allow them to distinguish the light reflections determined by differently sized supports. Alternatively, Souza and colleagues introduced the concept of “plant electrome”, describing the totality of the ionic dynamics at different scales of plant organization, engendering a constant electrical activity [34,35]. Souza and colleagues demonstrated that, rather than pure random noise, the amount of complexity characterizing environmental stimuli might alter several characteristics of the temporal dynamics of the plant electrome [34,36,37]. It was reported that some frequencies (the higher ones) exhibited by non-stimulated plants faded after stimulation. Only the lowest frequencies remain, allowing for low-energy-cost long-distance signaling [35]. In this view, the electrome could be considered a unifying factor of whole plant reactivity in a constantly changing environment and, therefore, might be a good candidate for understanding the flexible behavior of plants [35].

A caveat of the present results at the observational level is that the direction of the circular movements could be either clockwise or counterclockwise, and it could change within the same plant. Whether climbing plants are right- or left-handers is an aspect tackled in the previous literature [38], and that may be pursued in connection with decision-making. Further research is required to establish such a link.

In conclusion, the results of this study offer a contextual framework for the different well-known responses of climbing plants when searching for a support. More importantly, we have demonstrated a decision-making ability in plants, which allows them to adaptively ‘choose’ between responses according to the diameter of the available supports. Overall, the results of our study suggest that plants are capable of acquiring and integrating complex information about their environment in order to modify their extent of plastic responses adaptively. Such complex decision-making in plants could have important implications for our understanding of the processes that govern plant behavior.

## 4. Materials and Methods

### 4.1. Subjects

A total of 17 snow peas (*Pisum sativum* var. *saccharum* cv Carouby de Maussane) were chosen as study plants. Cylindrical pots (40 cm in diameter, 20 cm in depth) were filled with river sand (type 16SS, dimension 0.8/1.2 mm, weight 1.4). Seeds were potted at 8 cm from the pot’s border and sowed at a depth of 2.5 cm.

### 4.2. Type of Support

**Figure 3:**
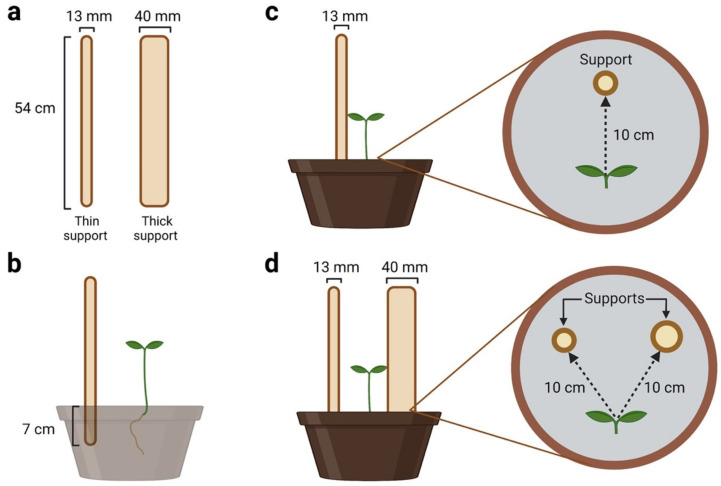
Graphical depiction of the (**a**) “thin” and “thick” supports; (**b**) the location of the support in the pot and how it was inserted in the soil. The single-support and double-support conditions are represented in panels (**c**) and (**d**), respectively.

Two types of wooden support were considered: a ‘thin’ support of 13 mm in diameter (Koto -13 mm) and a ‘thick’ support of 40 mm in diameter (Koto -40 mm; Figure 3a). Both supports were 54 cm in height. The supports were inserted 7 cm below the soil surface (Figure 3b). The supports were made available to the plants immediately after germination.

### 4.3. Experimental Conditions

The subjects were randomly assigned to two experimental conditions termed single- (SS) and double-support (DS) conditions. For the SS condition, 8 plants were raised individually in the presence of the ‘thin’ support (Figure 3c). For the DS condition (Figure 3d), 8 plants were raised individually in the presence of both the ‘thin’ and the ‘thick’ support. The location of the differently sized supports was counterbalanced across subjects to avoid a potential bias due to the direction of circumnutation (clockwise or counterclockwise). The supports were positioned so that the first leaf developed by a sprout faced the midpoint between the two supports. This was done to prevent a growing bias in favor of either one or the other support. It should be noted that here, we did not include a ‘thick’ single-support condition. This decision was based on the observation that, during data acquisition for the DS condition, none of the plants successfully touched or grasped the thick support—they all went for the thin support. Consequently, it would be impossible to compare trials for a potentially thick SS condition with trials for the DS condition. Moreover, the differences between the thin and thick supports have been previously reported [6,7,8], and it has been established that the thicker support is not the best option for climbing plants [4,11,12,13,14,15]. Therefore, we confined our comparison to plants that achieved the same outcome of touching and grasping the thin support under the SS and DS conditions.

In addition, our setting considered an equal distance between the plant and the surface of the supports and not necessarily the center of the support (Figure 3c,d). This appears to be a suitable positioning solution, given that we are focusing on the approaching phase preceding the grasping of the support and not on the coiling phase of the support. Note, however, that in the studies concerned with the measurements related to the coiling pattern, the equal distance between the plant and the exact center of the support has been considered [5].

### 4.4. Experimental Setup

**Figure 4:**
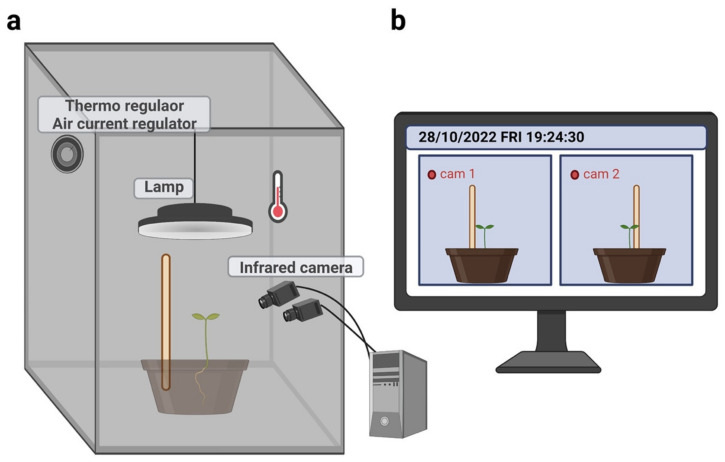
Graphical illustration of (**a**) experimental setup and (**b**) demonstration of how plants were captured by the infrared cameras.

The plants grew individually in a thermo-light-controlled growth chamber (Cultibox SG combi 80 × 80 × 160 cm; Figure 4). The temperature was set at 26 °C by means of an extractor fan equipped with a thermo-regulator (TT125 vents; 125 mm-diameter; max 280 mc/h) and an input-ventilation fan (Blauberg Tubo 100–102 m^3^/h). The two-fan combination allowed for a steady air flow rate into the growth chamber with a mean air residence time of 60 s. The fan was carefully placed so that the circulation of air did not affect the plants’ movements. Each plant was exposed for 12 h (6 a.m. to 6 p.m.) to a cool white LED lamp (V-TAC innovative LED lighting, VT-911-100W, Des Moines, IA, USA) that was positioned 50 cm above each seedling. The photosynthetic photon flux density at 50 cm under the lamp in correspondence with the seedling was 350 µmol_ph_/(m^2^s) (quantum sensor LI-190R, Lincoln, NE, USA). At the beginning of each experiment, the pots were fertilized using a half-strength solution culture (Murashige and Skoog Basal Salt Micronutrient Solution; see https://www.sigmaaldrich.com/RO/en/technical-documents/technical-article/cell-culture-and-cell-culture-analysis/plant-tissue-culture/murashige-skoog, accessed on 20 February 2023). The pots were watered with 1 L a week using distilled water (Sai Acqua Demineralizzata, Parma, Italy).

### 4.5. Kinematic Acquisition and Data Processing

For each growth chamber, a pair of RGB-infrared cameras (IP 2.1 Mpx outdoor varifocal IR 1080P) were placed 110 cm above the ground, spaced at 45 cm to record the stereo images of the plant (Figure 4). The cameras were connected via ethernet cables to a 10-port wireless router (D-Link Dsr-250n) connected via Wi-Fi to a PC. The frame acquisition and saving processes were controlled by CamRecorder software (Ab.Acus s.r.l., Milan, Italy; Figure 4). Each camera’s intrinsic, extrinsic, and lens distortion parameters were estimated using a Matlab Camera Calibrator application. Depth extraction from the single images was carried out by taking 20 pictures of a chessboard (squares’ size of 18 × 18 mm, 10 columns × 7 rows) from multiple angles and distances in natural non-direct light conditions. For the stereo calibration, the same chessboard used for the single-camera calibration process was placed in the middle of the growth chamber. The two cameras synchronously acquired the frame every 180 s (frequency 0.0056 Hz). RGB images were acquired during the daylight cycle, and infrared images during the night cycle. The anatomical landmarks of interest were the tendrils developing from the considered leaf. We considered the initial frame as the one corresponding to the appearance of the tendrils for the considered leaf. The end frame was defined as the frame in which the tendrils start to coil the support. Images from both the left and right cameras were used in order to reconstruct 3D trajectories. An ad hoc software (Ab.Acus s.r.l., Milan, Italy), developed in Matlab, was used to identify the anatomical points to be investigated by means of markers and to track their position frame-by-frame on the images acquired by the two cameras to reconstruct the 3D trajectory of each marker. The markers on the anatomical landmarks of interest (i.e., the tendrils) were inserted post hoc. The tracking procedures were performed automatically throughout the time of the movement sequence using the Kanade–Lucas–Tomasi (KLT) algorithm on the frames acquired by each camera after distortion removal. The tracking was manually verified by the experimenter, who checked the position of the markers frame-by-frame. The 3D trajectory of each tracked marker was computed by triangulating the 2D trajectories obtained from the two cameras. Finally, the trajectory was reconstructed with a series of coordinates in 3D (x, y, z), where the x-z plane is the horizontal plane, and the x-y plane and z-y plane are the vertical planes perpendicular to each other.

### 4.6. Dependent Measure

**Figure 5:**
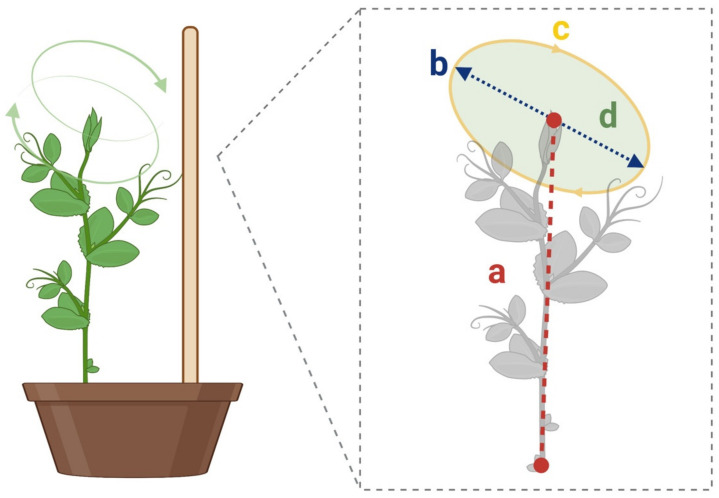
Graphical representation for some of the considered dependent measures: (**a**) the distance from the circumnutation gravity center to the origin is represented as a red/dash line; (**b**) the length of the circumnutation major axis is represented as a blue/dash line; (**c**) the circumnutation length is represented as a yellow/solid line; (**d**) the circumnutation area is represented in green.

The considered dependent measures were the following [39]:

(i) The number of circumnutations: the number of circumnutations performed by a plant from the time it was potted to the time it touched the support.
(ii) The circumnutation duration: the time taken by a plant to complete a single circumnutation.
(iii) Distance from the circumnutation gravity center to the origin (Figure 5. Segment a): the distance between the circumnutation gravity center and the plant origin.
(iv) The length of the circumnutation major axis (Figure 5. Segment b): the maximum distance between two points of the circumnutation trajectory.
(v) The circumnutation length (Figure 5. Segment c): the length of the overall path computed as the sum of all the Euclidean distances between the subsequent points during a single circumnutation.
(vi) The circumnutation area (Figure 5. Segment d): the sum of pixels with a value equal to 1, obtained from the binarization of the circumnutation trajectory.
(vii) The amplitude of peak velocity: the values for the average of the maximum velocity.

### 4.7. Statistical Analysis

The descriptive statistics, including the mean, standard deviation (SD), standard error (SE), and coefficient of variation, were calculated. Statistical analyses were conducted using the Bayesian approach. The objective of Bayesian estimation is to allocate credibility to a distribution of alternative parameter values (posterior distribution) that is consistent with the observed data by generating a large number of samples using the Markov chain Monte Carlo approach (MCMC). In this study, we adopted the two-sided Bayesian Mann–Whitney U test, given that the dependent variables are not normally distributed. The Mann–Whitney U test is a non-parametric test that does not require the assumption of normality. The analysis was performed using JASP [40], which was nested within the environment R (see the https://jasp-stats.org/r-package-list/, accessed on 20 February 2023) [41]. We choose the default that was prior defined by a Cauchy distribution, which was centered on a zero-effect size (δ) and a scale of 0.707 because prior knowledge regarding the exposition of plants to a double-support condition is absent [42,43]. Data augmentation was generated with five chains of 1000 iterations, allowing for a simpler and more feasible simulation from a posterior distribution. In the analysis, *W* was calculated in the Mann–Whitney U test as the smaller of the rank total between the two conditions. The Bayes factor (BF) was obtained to quantify the relative predictive performance of two hypotheses [42]. The BF quantifies evidence for the presence or absence of the difference between the DS and SS conditions. Here, the null hypothesis (H0) is that there is no difference in kinematics between the DS and SS conditions. The alternative hypothesis (H_1_) is that there is a difference. The BF_10_ value is the likelihood given H_1_ divided by H_0_. The BF_01_ value is calculated as H_0_ divided by H_1_. The results are reported based on Jeffery’s scheme, which proposes a series of labels for which specific Bayes factor values can be considered as either “no evidence (0–1)”, “anecdotal (1–3)”, “moderate (3–10)”, “strong (10–30)”, “very strong (30–100)”, or “decisive (>100)” relative evidence for alternative hypotheses [44]. R-hat is also reported to check the degree of convergence of the MCMC algorithms based on outcome stability. The closer the value of R-hat is to 1, the better convergence to the underlying distribution. Credible intervals (CI) are set as 95%, which is simply the central portion of the posterior distribution that contains 95% of the values.

## Figures and Tables

**Table 1:** Descriptive statistics for the considered dependent measures.

	Group	Mean	SD	SE	Coefficient of Variation	95% CI	
						Lower	Upper
Number of circumnutations	DS	24.924	4.247	0.303	0.170	24.327	25.521
	SS	26.553	6.156	0.439	0.232	25.688	27.418
Circumnutation duration (min)	DS	66.746	13.190	0.940	0.198	64.893	68.600
	SS	69.000	14.451	1.030	0.209	66.969	71.031
Distance from the circumnutation gravity center to the origin (cm)	DS	15.899	10.429	0.743	0.656	14.434	17.364
	SS	27.895	24.340	1.734	0.873	24.475	31.315
Length of the circumnutation major axis (mm)	DS	91.214	38.929	2.774	0.427	85.744	96.684
	SS	72.908	43.538	3.102	0.597	66.791	79.026
Circumnutation length (mm)	DS	243.403	124.957	8.903	0.513	225.846	260.961
	SS	188.148	115.972	8.263	0.616	171.853	204.443
Circumnutation area (mm^2^)	DS	4992.504	4634.422	330.189	0.928	4341.325	5643.684
	SS	3217.099	3505.097	249.728	1.090	2724.601	3709.598
Amplitude of maximum peak velocity (mm/min)	DS	6.541	5.650	0.403	0.864	5.748	7.335
	SS	4.660	2.840	0.202	0.610	4.260	5.059

Note. DS = double-support condition; SS = single-support condition; SD = standard deviation; SE = standard error; CI = credible interval.

**Table 2:** Two-sided Bayesian Mann–Whitney U test for the DS and the SS conditions.

	BF₁₀	*W*	R-Hat
Number of circumnutation	314.656	14,220.000	1.008
Circumnutation duration	0.387	17,083.000	1.000
Distance from the circumnutation gravity center to the origin	136.096	15,132.000	1.031
Length of the circumnutation major axis	734.705	24,455.000	1.016
Circumnutation length	980.421	24,433.000	1.015
Circumnutation area	1267.886	24,611.500	1.008
Amplitude of maximum peak velocity	4137.588	25,438.000	1.014

Note. Results based on data augmentation algorithm with five chains of 1000 iterations.

## Data Availability

The data is available online: http://doi.org/10.6084/m9.figshare.22574443, accessed on 20 February 2023.

## Supplementary Materials

The following supporting information can be downloaded at: https://www.mdpi.com/article/10.3390/plants12081597/s1, Video S1: Plant in DS condition.
